# Association of Systemic Inflammation with Nocturnal Sleeping Time Among Terminally Ill Patients with Cancer: Preliminary Findings

**DOI:** 10.3390/healthcare13222959

**Published:** 2025-11-18

**Authors:** Koji Amano, Kengo Imai, Saori Toyota, Toshihiro Yamauchi, Satoru Miwa, Misuzu Yuasa, Soichiro Okamoto, Satoshi Inoue, Takamasa Kogure, Tatsuya Morita

**Affiliations:** 1 Department of Supportive and Palliative Care, Osaka International Cancer Institute, 3-1-69 Otemae, Chuo-ku, Osaka 541-8567, Japan; 2 Seirei Hospice, Seirei Mikatahara General Hospital, 3453 Mikatahara-cho, Chuo-ku, Hamamatsu 433-8558, Japan; k.imai@sis.seirei.or.jp (K.I.); t-yamauchi@sis.seirei.or.jp (T.Y.); s.miwa@sis.seirei.or.jp (S.M.); 3 Paramount Bed Sleep Research Laboratory, Paramount Bed Co., Ltd., 2-14-5 Higashisuna, Koto-ku, Tokyo 136-8670, Japan; s.toyota@paramount.co.jp (S.T.); t.kogure@paramount.co.jp (T.K.); 4 Department of Palliative Medicine, Suzuka General Hospital, 1275-53 Yamanohana, Yasuzuka-cho, Suzuka 513-8630, Japan; misuzu.yuasa@miekosei.or.jp; 5 Uguisu Home Clinic, 3-4-17 Nakajima, Naka-ku, Hamamatsu 430-0856, Japan; contact@uguisu-zaitaku.com; 6 Clinical Laboratory Department, Seirei Mikatahara General Hospital, 3453 Mikatahara-cho, Chuo-ku, Hamamatsu 433-8558, Japan; inosa@sis.seirei.or.jp; 7 Department of Palliative and Supportive Care, Seirei Mikatahara General Hospital, 3453 Mikatahara-cho, Chuo-ku, Hamamatsu 433-8558, Japan; tmorita@sis.seirei.or.jp; 8 Research Association for Community Health, 3-24-2 Somechidai, Hamana-ku, Hamamatsu 434-0046, Japan

**Keywords:** cancer, cachexia, systemic inflammation, C-reactive protein, sleep, palliative care

## Abstract

**Objectives:** Evidence regarding the impacts of systemic inflammation on nocturnal sleep in advanced cancer patients is limited. We determined the association of serum C-reactive protein (CRP) levels with sleep in patients with non-imminent and those with imminent death. **Methods:** This was a secondary analysis of an observational study conducted in patients newly referred to a palliative care unit. Nocturnal sleep was assessed based on the “sleeping time” measured using a sheet-type non-wearable sensor. Patients were divided into long-survival and short-survival groups depending on the median survival (11 days), and within each group, the patients were categorized according to CRP levels: low (<1 mg/dL), moderate (1–10 mg/dL), and high (≥10 mg/dL). To evaluate correlations between CRP levels and sleeping time, binomial logistic analysis was performed. Adjusted odds ratios (ORs) and 95% confidence intervals (CIs) were calculated. **Results:** A total of 535 patients were included in the main analysis. In the long-survival group (n = 273), the high-CRP patients had significantly longer sleeping time than the low-CRP patients (OR 2.81, 95% CI 1.19–6.65, *p*-value 0.019), whereas there were no significant correlations in the short-survival group (n = 262). **Conclusions:** Higher CRP levels were associated with longer sleeping time in patients with non-imminent death, whereas there were no correlations in patients whose death was imminent. The clinical implications of serum CRP levels appear to vary with life expectancy in terminally ill patients with cancer. Further research is necessary to verify the present findings.

## 1. Introduction

Crosstalk between tumor and host immune system produces pro-inflammatory cytokines, leading to systemic inflammation [1,2,3,4,5,6]. Evidence suggests that systemic inflammation is involved in the mechanisms responsible for cancer cachexia [1,2,3,4,5,6]. In patients with cancer cachexia, pro-inflammatory cytokines are amplified through alterations in the central nervous system (CNS), particularly the hypothalamic–pituitary–adrenal (HPA) axis, causing sympathetic nerve dominance [7,8,9,10]. In addition, activation of the HPA axis and sympathetic nerve dominance provoke muscle atrophy and sleep disorders and insufficiencies through the secretion of glucocorticoids by the adrenal gland [7,11]. Thus, systemic inflammation generates a variety of physical and psychological symptoms in individuals with advanced cancer. Multiple events appear to be induced by CNS inflammation in individuals affected by cancer cachexia [12].

Previous studies reported that pain and symptoms have frequently been detected in cancer patients with systemic inflammation [13,14,15,16,17,18,19]. Moreover, psychological symptoms are more frequently observed in cachectic patients with cancer than in non-cachectic patients [20]. Furthermore, serum C-reactive protein (CRP) levels have been considered as a surrogate of systemic inflammation, which was associated with survival, daily activities, and physical and psychological symptoms in individuals with incurable advanced cancer in palliative care settings [15,19,21,22,23,24,25,26].

However, there is insufficient evidence to show that systemic inflammation contributes to the genesis of psychological symptoms through alterations in the CNS/HPA axis in patients with advanced cancer [12,20]. Moreover, there is limited evidence regarding the negative impacts of systemic inflammation on the status of night sleep in this population, although sleep disorders and insufficiencies are associated with activation of the HPA axis and sympathetic nerve dominance [11]. Furthermore, no reports have shown whether the clinical implications of serum CRP levels vary with life expectancy in patients with advanced cancer.

Therefore, we determined the association of serum CRP levels with night sleep (i.e., total sleeping time and restlessness were measured using a sheet-type non-wearable sensor) in non-imminent death and imminent death patients with advanced cancer who were admitted to a palliative care unit. Furthermore, we also investigated the relationship between serum CRP levels and the severity of symptoms, drug use (opioid oral morphine, benzodiazepine, and/or psychotropic drugs), and the patients’ delirium.

## 2. Participants and Methods

### 2.1. Sites and Participants

Herein, a secondary analysis of an observational study with a retrospective design was performed. The study was conducted in a palliative care unit with 27 beds (located in a cancer-designated hospital with 940 beds) in Japan between March 2019 and December 2022. The aim of the study was to determine the effectiveness of a sheet-type non-wearable sensing device in terminally ill patients with cancer, who had a survival of days to weeks [27].

All patients meeting the eligibility criteria were enrolled. The inclusion criteria were (1) patients admitted to a palliative care unit, (2) patients aged ≥18 years, and (3) patients diagnosed with locally advanced or metastatic cancer. The exclusion criteria were (1) patients who were scheduled to be discharged within one week, (2) patients or family members who refused to participate in the study, (3) poor patient condition that did not allow for transfer to the bed with a Nemuri SCAN device, and (4) patients who were discharged alive.

### 2.2. Measurements

The status of night sleep was assessed based on the sleeping time and activity scores measured using a sheet-type non-wearable sensor (length, 28.4 cm; width, 77.5 cm; thickness, 1.8 cm), namely a Nemuri SCAN device (Paramount Bed Co., Ltd., Tokyo, Japan). The highly sensitive pressure sensor is placed under a mattress and detects the movement of patients lying on top of the mattress. Nemuri SCAN identifies whether a subject is asleep or awake using movement data to measure total sleeping time. Nemuri SCAN also analyses the intensity and frequency of body movements every minute, which are recorded as activity scores, excluding breathing and heartbeats. Activity scores range from 0 to 960 scores/min because data are sampled at 16 Hz (16 times per second). Average activity scores (i.e., scores/minute) are used for analyses. The accuracy of these assessments has been validated, showing that these parameters can serve as objective measurements of the status of night sleep. Activity scores evaluated using a sheet-type sensor can assess not only agitation but also sedation levels [27,28].

A calibrated version of the Nemuri SCAN device was used in the study. We set up Nemuri SCAN under the participants’ bed mattresses and obtained data on their sleeping or awake status and activities every minute for 24 h from the day of admission to the day of discharge. The data were recorded on a storage medium built into the device. Data on patient sleeping status (i.e., total sleeping time and activity score) were extracted from the device by one of the researchers (ST), who was blinded to the clinical data. We exclusively used Nemuri SCAN data obtained during the night (23:00–6:00) for this study.

Physicians in the palliative care unit extracted data on patient characteristics from the medical records upon admission. They also collected the results of blood tests performed within three days after admission. When no data on blood was collected, physicians recorded the latest blood test results obtained before death. They also retrieved data on the severities of symptoms (i.e., pain, dyspnea, fatigue, and nausea) assessed using the Integrated Palliative care Outcome Scale (IPOS) [29,30], opioid oral morphine milligram equivalent based on the Clinical Guidelines from the Japanese Society of Palliative Medicine [31], presence of benzodiazepine and/or psychotropic drugs, and scores of the modified Richmond Agitation–Sedation Scale (mRASS) [32,33] from three days, one week, two weeks, three weeks, and four weeks prior to a patient’s death. The mRASS scores were recorded by nurses in the palliative care unit three times per day (i.e., 8:30–17:00, 17:00–1:00, and 1:00–8:30), documented on the medical record within the routine practice. Both the IPOS and mRASS were translated into Japanese, which were validated in patients with advanced cancer in palliative care settings [34,35]. The nurses were blinded to the Nemuri SCAN data of participating patients.

A database for this secondary analysis was created to pair the blood test results with Nemuri SCAN data obtained during the night (23:00–6:00) on the same day. Furthermore, data on symptoms, opioid oral morphine milligram equivalent, benzodiazepine and/or psychotropic drugs, and mRASS scores obtained within three days of the given day were considered.

We hypothesized that total sleeping time shortens and restlessness worsens as systemic inflammation becomes more severe in patients with imminent death compared to those with non-imminent death.

### 2.3. Statistical Analysis

Patient characteristics were presented as numbers (%) or medians (interquartile range), where appropriate. Participants were divided into three groups according to their serum levels of CRP: (1) low (CRP levels < 1 mg/dL), (2) moderate (1 ≤ CRP levels < 10 mg/dL), and (3) high (CRP levels ≥ 10 mg/dL). These cutoffs were predefined based on a clinical rationale and previous clinical research [15,19,36,37]. Comparisons among the three categories were made using the Kruskal–Wallis test or chi-squared test, where appropriate.

To examine differences in the clinical implications of CRP levels based on survival time, patients above and below the median survival (11 days) were divided into two groups because of no validated cutoff value: long-survival group (≥11 days) and short-survival group (≤10 days). After that, to evaluate correlations between CRP levels and sleeping time and associations between CRP levels and activity scores in both the long- and short-survival groups, binomial logistic analysis was performed. Dependent variables (i.e., sleeping time and activity scores) were divided into two groups using their respective median values, because no appropriate cutoff values have been shown. Adjusted odds ratios (ORs) and 95% confidence intervals (CIs) were calculated after adjusting for independent variables considered to be potential risk factors for affecting night sleep in patients with advanced cancer, including age, sex, primary tumor site, opioid oral morphine milligram equivalent (per 10 mg), benzodiazepine and/or psychotropic drugs (yes or no), and CRP level (three categories). We entered these variables into the logistic regression models because they were considered potential confounding variables [19].

All results were considered to be significant if the *p*-value was less than 0.05. All analyses were performed using SPSS software version 22.0.

## 3. Results

**Figure 1:**
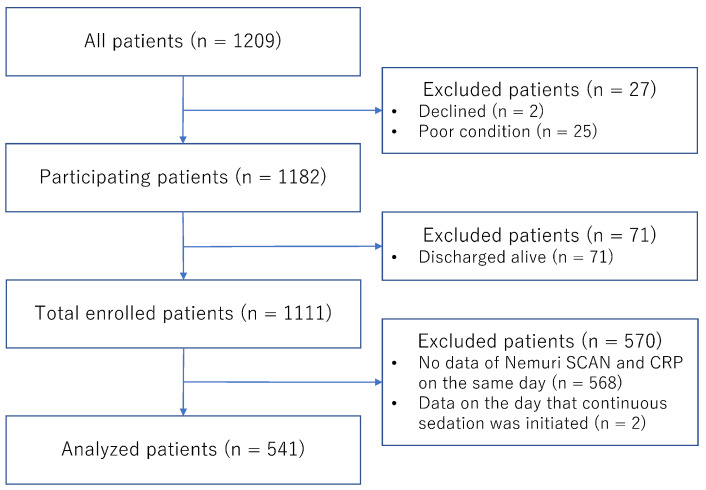
Study diagram. CRP, C-reactive protein.

A total of 1209 patients were admitted to the palliative care unit during the observation period. Among them, 2 declined consent, 25 were excluded due to technical issues (e.g., unable to be transferred to the bed with Nemuri SCAN because of their poor condition), and 71 were discharged alive. Thus, a total of 1111 patients were enrolled in the original study. Of these patients, 568 were excluded due to a lack of Nemuri SCAN and CRP data on the same day, and 2 were excluded because data were obtained on the day that continuous sedation was initiated. Finally, 541 patients were included in this secondary analysis (Figure 1).

Patient characteristics are presented in Table 1. The median age was 75 years old. The proportion of males was 55.8%. The top three sites of primary cancer were the upper and lower gastrointestinal tracts (24.8%), liver, biliary system, and pancreas (21.8%), and lung (21.1%). Furthermore, 42.0% of the patients had lung metastases, and 37.2% had liver metastases.

According to their serum CRP levels, the 541 patients were categorized as low (n = 66), moderate (n = 290), and high (n = 185). Relationships between CRP levels, symptoms, and other variables in all patients are shown in Table 2. Higher CRP levels were significantly associated with more severe pain and more opioid use (*p* = 0.014 and *p* < 0.001, respectively). Moreover, relationships between CRP levels and Nemuri SCAN parameters in all patients are shown in Table 3. The higher the CRP levels, the longer the sleeping time and the lower the activity scores appeared to be, but no significant differences were found. However, higher CRP levels were correlated with significantly higher heart rates (*p* < 0.001).

**Figure 2:**
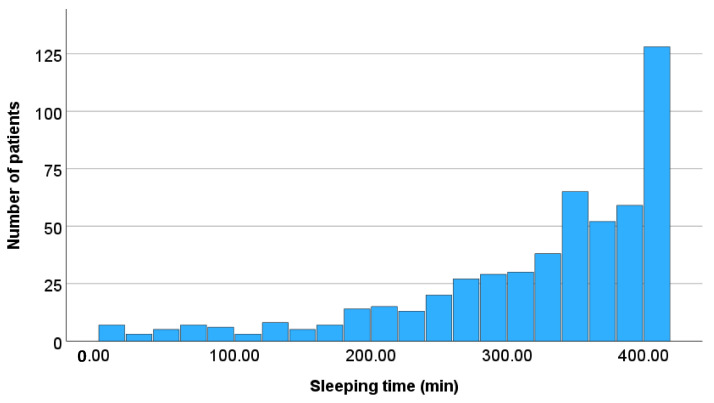
Relationship between the number of patients and daily sleeping time. As the sleeping time decreased, the number of patients decreased.

**Figure 3:**
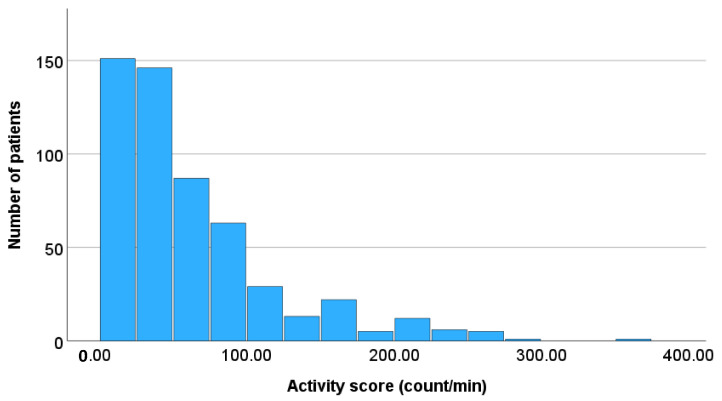
Relationship between the number of patients and daily activity scores. As the activity scores increased, the number of patients decreased.

The relationship between the number of patients and sleeping time, and the relationship between the number of patients and activity scores in all patients are illustrated in Figure 2 and Figure 3, respectively. As the sleeping time decreased, the number of patients decreased. Furthermore, as the activity scores increased, the number of patients decreased.

Results of the multivariate logistic regression analysis are presented in Table 4, showing the associations between CRP levels and sleeping time in the long-survival group (n = 273) and short-survival group (n = 262). Patients with high CRP levels (≥10 mg/dL) had significantly longer night sleeping time than those with low CRP levels (<1 mg/dL) in patients who survived more than 11 days (OR 2.81, 95% CI 1.19–6.65, *p*-value 0.019). However, there were no significant correlations in patients who survived less than 10 days.

Results of the multivariate logistic regression analysis are also presented in Table 5, showing the associations between CRP levels and activity scores in the long-survival group (n = 273) and short-survival group (n = 262). Patients with high CRP levels (≥10 mg/dL) tended to have less activity at night than those with low CRP levels (<1 mg/dL) in patients who survived more than 11 days (OR 0.46, 95% CI 0.20–1.08, *p*-value 0.076). However, there were no significant correlations in patients who survived less than 10 days.

## 4. Discussion

To date, there is limited evidence on the negative impacts of systemic inflammation on the status of night sleep in patients with advanced cancer. We determined the association of serum CRP levels with night sleep, considering survival time among terminally ill patients with cancer admitted to a palliative care unit. Our results showed that a higher CRP level (≥10 mg/dL) was significantly associated with longer sleeping time during the night in patients with longer survival time (≥11 days), but it was not significant for activity score during the night. Furthermore, there were no associations between CRP levels and sleeping time or activity scores in patients with shorter survival time (≤10 days). The findings implied that patients with non-imminent death were likely to be simply and highly affected by systemic inflammation, and that patients with impending death were bedridden regardless of systemic inflammation or delirious and restless due to factors other than systemic inflammation. However, these need to be verified in the future.

A previous study conducted on patients with advanced cancer admitted to palliative care units reported a significant association between a higher CRP level (≥10 mg/dL) and the occurrence of delirium [19], suggesting that patients with systemic inflammation tend to have restlessness due to delirium. Moreover, a prior study revealed that the activity scores reflecting restlessness measured using the Nemuri SCAN were significantly and positively associated with the levels of terminal agitation, or delirium [27]. Against our expectations, however, there was no association between CRP levels and activity scores in patients with impending death in this study. A secondary analysis of two randomized clinical trials conducted in palliative care units showed that cancer patients with end-of-life delirium were more restless between 15:00 and 23:00 [38], while this study used data obtained between 23:00 and 6:00. This may be one of the reasons why no associations were observed between CRP levels, sleeping time, and activity levels in patients in the dying phase.

Sickness behavior is considered a symptom cluster (e.g., fever, fatigue, drowsiness, nausea, anorexia, depression, anxiety, and cognitive impairment) caused by CNS inflammation, which is followed by systemic inflammation in animals [39]. CNS inflammation is directly induced by inflammatory mediators, such as pro-inflammatory cytokines, in individuals with sickness, and the CNS/HPA axis controls behavioral changes (e.g., decline in motivation, sleep disturbance, and cognitive impairment) through the immune system and the CNS in animals and humans [40]. Other phenotypes (e.g., increased catabolism, weight loss, and circadian rhythm disorders) are also related to sickness behavior through an integrated response of CNS inflammation and systemic inflammation [39,40]. Accordingly, there are many overlapping features between cancer cachexia and sickness behavior, and such phenomena are sustained and exacerbated by persistent CNS inflammation and systemic inflammation in patients with advanced cancer and those with infectious diseases. Hence, cancer cachexia may be understood in the context of sickness behavior, although there is limited evidence concerning the relationship between them. Clarifying this may be essential for explaining what cancer cachexia is and why we suffer from it [12]. In summary, there is an urgent need for further research to understand the underlying pathophysiology of systemic inflammation and its impact on the CNS/HPA axis and to develop effective treatments for physical and psychological symptoms, including anorexia, sleep disturbance, and delirium, among cachectic patients with cancer.

The present study has several strengths and limitations. The dataset was obtained from a large single-center prospective observational study, which contained robust clinical information and enabled an accurate analysis. However, this study was based on a secondary analysis, and approximately 50% of the original cohort was excluded. The main reason for this was that blood tests were not frequently performed in palliative care units. In addition, a database for this study was created to pair the blood test results with Nemuri SCAN data on the same day. Moreover, the findings of this study demonstrated that higher CRP levels were significantly associated with longer night sleeping time in patients with non-imminent death. However, the association can be reverse causal, or even bidirectional. This study did not examine the causes of elevated CRP levels, but it investigated the association of serum CRP levels with sleep disturbance. A serum CRP level is not a specific marker, but the clinical significance of CRP remains unchanged regardless of the reason for elevated CRP levels. According to previous studies, elevated CRP levels are undoubtedly a poor prognostic factor, regardless of their cause [15,19,21,22,23,24,25,26]. The causes are often complex and coexist (e.g., cancer cachexia, acute infection, chronic heart failure, and obesity), and it is difficult to detect a single factor in clinical practice. Additionally, it was impossible to assess other factors that may influence the associations between CRP and sleeping time or activities because potential confounders could not be identified. To investigate the causal mechanisms of serum CRP levels and sleep disturbance, it is necessary to stratify by CRP levels and adjust for potential confounders, including acute infection and chronic heart failure, in clinical studies. Changes in CRP levels are also very important in clinical practice. The survival cutoff value of 11 days has not been validated, but dividing patients receiving palliative care into two groups using ≥11 days and ≤10 days seems to be clinically acceptable. Furthermore, it was difficult to differentiate between a participant who was truly sleeping and one who was under some level of sedation induced by medication, including morphine, benzodiazepines, and psychotropic drugs, using the sleeping time and activity scores measured using just a sheet-type non-wearable sensor. The effectiveness of anti-inflammatory drugs and pain and symptom management in improving sleep status must be examined in the future. The results obtained should be confirmed in prospectively derived data on the patient cohort of interest who have complained of sleep disturbance, with appropriate outcomes assessed using objective and subjective parameters to translate research findings into clinical practice.

## 5. Conclusions

A higher CRP level was significantly associated with longer sleeping time during the night in patients whose death was less imminent, but it was not significant for activity score during the night. Furthermore, there were no correlations in patients whose death was imminent. The clinical implications of serum CRP levels appear to vary with life expectancy in terminally ill patients with cancer. Further research is necessary to verify the present findings and clarify their relevance in clinical assessment and management of sleep-related symptoms in palliative care practice.

## Figures and Tables

**Table 1:** Patient Characteristics (n = 541).

Age in years	75 (69, 82)
Sex	
Male	302 (55.8)
Female	239 (44.2)
Primary cancer site	
Upper and lower gastrointestinal tracts	134 (24.8)
Liver, biliary system, and pancreas	118 (21.8)
Lung	114 (21.1)
Urological	42 (7.8)
Gynecologic	26 (4.8)
Breast	25 (4.6)
Hematological	16 (3.0)
Others	66 (12.2)
Metastasis	
Lung	227 (42.0)
Liver	201 (37.2)
Bone	117 (21.6)
Central nervous system	67 (12.4)

Values represent n (%) or the median (interquartile range).

**Table 2:** Relationships between CRP levels and symptoms, including other variables.

	Total (n = 541)	CRP < 1 (n = 66)	1 ≤ CRP < 10 (n = 290)	10 ≤ CRP (n = 185)	*p*-Value
Symptom, Integrated Palliative Care Outcome Scale (0–4)					
Pain	1 (0, 1)	0 (0, 1)	0 (0, 1)	1 (0, 1)	0.014
Dyspnea	0 (0, 1)	0 (0, 0)	0 (0, 1)	0 (0, 1)	0.067
Fatigue	1 (1, 1)	1 (0, 1)	1 (1, 1)	1 (1, 1)	0.096
Nausea	0 (0, 0)	0 (0, 0)	0 (0, 0)	0 (0, 0)	0.234
Opioid oral morphine milligram equivalent (mg/day)	24.0 (0.0, 60.0)	10.6 (0.0, 30.0)	24.0 (0.0, 48.0)	24.0 (10.0, 84.0)	<0.001
Benzodiazepine and/or psychotropic drug	291 (53.8)	35 (53.0)	142 (49.0)	114 (61.6)	0.022
Modified Richmond Agitation-Sedation Scale (−5 to +4)	0 (0, 0)	0 (0, 0)	0 (0, 0)	0 (0, 0)	0.480
Survival time (days)	11.0 (6.0, 19.0)	13.0 (6.8, 21.0)	11.0 (6.0, 20.0)	10.0 (5.0, 16.0)	0.079

Values represent n (%) or the median (interquartile range). Comparisons among the groups were made using the Kruskal–Wallis test or chi-squared test. CRP, C-reactive protein.

**Table 3:** Relationships between CRP levels and Nemuri SCAN parameters.

	Total (n = 541)	CRP < 1 (n = 66)	1 ≤ CRP < 10 (n = 290)	10 ≤ CRP (n = 185)	*p*-Value
Total sleeping time (min/day)	350.0 (275.0, 397.0) 321.5 ± 96.9	340.5 (265.3, 384.3) 314.0 ± 95.7	347.5 (264.5, 398.3) 319.1 ± 98.0	352.0 (301.5, 398.5) 327.8 ± 95.8	0.487
Activity score (count/min)	45.1 (22.5, 81.5) 61.6 ± 56.5	54.5 (22.5, 88.1) 65.1 ± 53.8	45.8 (22.2, 87.8) 63.1 ± 58.7	41.2 (22.6, 71.6) 58.1 ± 53.9	0.556
Time away from bed (min)	0.0 (0.0, 11.0) 10.4 ± 23.1	0.0 (0.0, 13.5) 9.0 ± 13.8	0.0 (0.0, 11.3) 11.2 ± 24.4	0.0 (0.0, 9.0) 9.7 ± 23.6	0.620
Number of times away from bed (count)	0.0 (0.0, 2.0) 1.4 ± 2.4	0.0 (0.0, 2.0) 1.2 ± 1.5	0.0 (0.0, 2.0) 1.4 ± 2.5	0.0 (0.0, 2.0) 1.3 ± 2.4	0.643
Respiratory rate (count/min)	13.9 (11.9, 16.4) 14.5 ± 3.3	14.0 (11.8, 16.2) 14.3 ± 3.1	13.7 (11.7, 16.1) 14.2 ± 3.1	14.1 (12.1, 17.0) 14.9 ± 3.7	0.182
Heart rate (count/min)	83.1 (72.8, 94.6) 83.9 ± 15.5	75.9 (67.2, 89.4) 77.8 ± 14.5	80.4 (71.3, 92.5) 81.4 ± 14.9	91.3 (78.8, 98.1) 90.0 ± 14.7	<0.001

Values represent the median (interquartile range) and mean ± standard deviation. Comparisons among the groups were made using the Kruskal–Wallis test. CRP, C-reactive protein.

**Table 4:** Association between CRP levels and total sleeping time.

	Long-Survival Group (n = 273)		Short-Survival Group (n = 262)	
	Adjusted OR (95% CI)	*p*-Value	Adjusted OR (95% CI)	*p*-Value
CRP (mg/dL)				
CRP < 1	1.00 (reference)		1.00 (reference)	
1 ≤ CRP < 10	1.93 (0.87, 4.26)	0.104	0.79 (0.33, 1.89)	0.596
10 ≤ CRP	2.81 (1.19, 6.65)	0.019	0.94 (0.38, 2.32)	0.886
Age, years	1.02 (0.99, 1.04)	0.159	1.01 (0.98, 1.03)	0.709
Sex				
Male	1.00 (reference)		1.00 (reference)	
Female	1.29 (0.75, 2.22)	0.364	1.73 (0.98, 3.05)	0.060
Primary cancer site				
Lung	1.00 (reference)		1.00 (reference)	
Upper and lower gastrointestinal tracts	0.62 (0.28, 1.35)	0.226	0.86 (0.41, 1.79)	0.683
Liver, biliary system, and pancreas	1.12 (0.51, 2.47)	0.781	0.56 (0.26, 1.21)	0.143
Breast	2.30 (0.59, 8.95)	0.230	0.43 (0.11, 1.65)	0.216
Gynecologic	1.26 (0.33, 4.78)	0.731	0.97 (0.25, 3.76)	0.965
Urological	1.23 (0.40, 3.75)	0.716	1.74 (0.62, 4.88)	0.293
Hematological	1.52 (0.30, 7.61)	0.610	1.80 (0.31, 10.34)	0.512
Others	0.47 (0.17, 1.30)	0.145	0.79 (0.35, 1.80)	0.571
Opioid oral morphine milligram equivalent (per 10 mg)	0.96 (0.92, 0.99)	0.024	0.98 (0.94, 1.01)	0.145
Benzodiazepine and/or psychotropic drug				
No	1.00 (reference)		1.00 (reference)	
Yes	0.84 (0.50, 1.43)	0.520	1.04 (0.61, 1.76)	0.896

Patients above and below the median survival (11 days) were separated into long-survival and short-survival groups. To evaluate the relationship between CRP levels and sleeping time, adjusted ORs and 95% CIs were calculated in the logistic regression models. The following variables were included as covariates: age, sex, primary tumor site, opioid oral morphine milligram equivalent, benzodiazepine and/or psychotropic drugs (yes or no), and CRP level. CRP, C-reactive protein; OR, odds ratio; CI, confidence interval.

**Table 5:** Association between CRP levels and activity scores.

	Long-Survival Group (n = 273)		Short-Survival Group (n = 262)	
	Adjusted OR (95% CI)	*p*-Value	Adjusted OR (95% CI)	*p*-Value
CRP (mg/dl)				
CRP < 1	1.00 (reference)		1.00 (reference)	
1 ≤ CRP < 10	0.66 (0.30, 1.45)	0.300	0.74 (0.31, 1.78)	0.507
10 ≤ CRP	0.46 (0.20, 1.08)	0.076	0.65 (0.26, 1.61)	0.351
Age, years	0.99 (0.97, 1.02)	0.510	1.00 (0.97, 1.02)	0.746
Sex				
Male	1.00 (reference)		1.00 (reference)	
Female	0.77 (0.45, 1.35)	0.364	0.80 (0.45, 1.43)	0.450
Primary cancer site				
Lung	1.00 (reference)		1.00 (reference)	
Upper and lower gastrointestinal tracts	1.68 (0.77, 3.67)	0.195	1.57 (0.74, 3.34)	0.240
Liver, biliary system, and pancreas	0.97 (0.44, 2.14)	0.932	3.17 (1.44, 6.97)	0.004
Breast	0.95 (0.25, 3.58)	0.938	6.70 (1.50, 29.98)	0.013
Gynecologic	0.80 (0.21, 3.09)	0.749	2.43 (0.62, 9.49)	0.200
Urological	1.03 (0.34, 3.13)	0.960	0.86 (0.30, 2.50)	0.786
Hematological	0.66 (0.13, 3.39)	0.623	0.77 (0.13, 4.48)	0.773
Others	3.44 (1.17, 10.14)	0.025	1.66 (0.72, 3.83)	0.238
Opioid oral morphine milligram equivalent (per 10 mg)	1.05 (1.01, 1.10)	0.016	1.05 (1.01, 1.09)	0.018
Benzodiazepine and/or psychotropic drug				
No	1.00 (reference)		1.00 (reference)	
Yes	1.73 (1.02, 2.94)	0.044	0.87 (0.50, 1.49)	0.608

Patients above and below the median survival (11 days) were separated into long-survival and short-survival groups. To evaluate the relationship between CRP levels and activity scores, adjusted ORs and 95% CIs were calculated in the logistic regression models. The following variables were included as covariates: age, sex, primary tumor site, opioid oral morphine milligram equivalent, benzodiazepine and/or psychotropic drugs (yes or no), and CRP level. CRP, C-reactive protein; OR, odds ratio; CI, confidence interval.

## Data Availability

The datasets generated and analyzed in the present study are not publicly available because sharing is not explicitly covered by patient consent.
